# *Gabra2* is a genetic modifier of Dravet syndrome in mice

**DOI:** 10.1007/s00335-021-09877-1

**Published:** 2021-06-04

**Authors:** Nicole A. Hawkins, Toshihiro Nomura, Samantha Duarte, Levi Barse, Robert W. Williams, Gregg E. Homanics, Megan K. Mulligan, Anis Contractor, Jennifer A. Kearney

**Affiliations:** 1 Department of Pharmacology, Northwestern University Feinberg School of Medicine, 320 E. Superior St., Searle 8-450, Chicago, IL 60611 USA; 2 Department of Physiology, Northwestern University Feinberg School of Medicine, Chicago, IL 60611 USA; 3 Department of Genetics, Genomics and Informatics, College of Medicine, University of Tennessee Health Science Center, Memphis, TN 38163 USA; 4 Department of Anesthesiology and Perioperative Medicine, Neurobiology, and Pharmacology and Chemical Biology, University of Pittsburgh, Pittsburgh, PA 15261 USA; 5 Department of Neurobiology Weinberg College of Arts and Sciences, Northwestern University, Evanston, IL 60208 USA

## Abstract

Pathogenic variants in epilepsy genes result in a spectrum of clinical severity. One source of phenotypic heterogeneity is modifier genes that affect expressivity of a primary pathogenic variant. Mouse epilepsy models also display varying degrees of clinical severity on different genetic backgrounds. Mice with heterozygous deletion of *Scn1a* (*Scn1a*^*+/−*^) model Dravet syndrome, a severe epilepsy most often caused by *SCN1A* haploinsufficiency. *Scn1a*^*+/−*^ mice recapitulate features of Dravet syndrome, including spontaneous seizures, sudden death, and cognitive/behavioral deficits. *Scn1a*^*+/−*^ mice maintained on the 129S6/SvEvTac (129) strain have normal lifespan and no spontaneous seizures. In contrast, admixture with C57BL/6J (B6) results in epilepsy and premature lethality. We previously mapped Dravet Survival Modifier loci (*Dsm1-Dsm5*) responsible for strain-dependent differences in survival. *Gabra2*, encoding the GABA_A_ α2 subunit, was nominated as a candidate modifier at *Dsm1*. Direct measurement of GABA_A_ receptors found lower abundance of α2-containing receptors in hippocampal synapses of B6 mice relative to 129. We also identified a B6-specific single nucleotide deletion within *Gabra2* that lowers mRNA and protein by nearly 50%. Repair of this deletion reestablished normal levels of *Gabra2* expression*.* In this study, we used B6 mice with a repaired *Gabra2* allele to evaluate *Gabra2* as a genetic modifier of severity in *Scn1a*^*+/−*^ mice. *Gabra2* repair restored transcript and protein expression, increased abundance of α2-containing GABA_A_ receptors in hippocampal synapses, and rescued epilepsy phenotypes of *Scn1a*^*+/−*^ mice. These findings validate *Gabra2* as a genetic modifier of Dravet syndrome, and support enhancing function of α_2_-containing GABA_A_ receptors as treatment strategy for Dravet syndrome.

**Supplementary Information:**

The online version contains supplementary material available at 10.1007/s00335-021-09877-1.

## Introduction

Dravet syndrome is a severe, infant-onset developmental and epileptic encephalopathy (DEE) with at least 80% of cases resulting from de novo pathogenic variants in *SCN1A* (Claes et al. 2001). Individuals with Dravet syndrome display multiple seizure types that are often refractory to standard therapeutics, an elevated risk for sudden unexpected death in epilepsy (SUDEP), cognitive and behavioral deficits, and motor system dysfunctions (de Lange et al. 2019; Dravet 2011). Phenotype heterogeneity is common in monogenic epilepsy syndromes, with a spectrum of clinical presentations, ranging from benign seizures to intractable epilepsy and increased SUDEP risk (Cetica et al. 2017; Escayg et al. 2000; Gardella et al. 2016; Goldberg-Stern et al. 2014; Helbig and Tayoun 2016; Møller et al. 2015; Nakamura et al. 2013; Patino et al. 2009; Syrbe et al. 2016; Weckhuysen et al. 2012). Modifier genes that affect penetrance and expressivity are likely contributors to this variability and may serve as effective therapeutic targets for epilepsy.

*SCN1A* haploinsufficiency is the major cause of Dravet syndrome; therefore, mice with heterozygous deletion of *Scn1a* were developed to model Dravet syndrome (Miller et al. 2014; Ogiwara et al. 2007; Yu et al. 2006). *Scn1a*^*+/−*^ mice recapitulate several core features of Dravet syndrome, including spontaneous seizures, elevated risk of sudden death, and neurobehavioral and cognitive deficits (Han et al. 2012; Hawkins et al. 2017a; Ito et al. 2013; Kalume et al. 2013; Miller et al. 2014; Ogiwara et al. 2013). These overt phenotypes are highly penetrant on the C57BL/6 J (B6) genetic background, but are absent when the mutation is on inbred 129 strain backgrounds (Kang et al. 2018; Miller et al. 2014; Ogiwara et al. 2007; Yu et al. 2006). Although 129.*Scn1a*^*+/−*^ mice do not exhibit spontaneous seizures, they have elevated susceptibility to acute seizures induced by hyperthermia or chemoconvulsants (Mistry et al. 2014; Rubinstein et al. 2015). Taking advantage of the strain difference, we previously performed genetic mapping and identified several Dravet survival modifier (*Dsm*) loci responsible for strain-dependent differences in incidence of sudden unexpected death (*Dsm1-5*) (Miller et al. 2014). *Gabra2*, encoding the α2 subunit of the GABA_A_ receptor, was the highest ranked candidate gene at the *Dsm1* locus (Hawkins et al. 2016; Miller et al. 2014). Notable differences in *Gabra2* expression among several inbred mouse strains had been previously reported (Korostynski et al. 2006; Mulligan et al. 2012; Yeo et al. 2016; Yu et al. 2020). Further work investigating a *Gabra2* expression quantitative trait locus (eQTL) identified a single nucleotide de novo deletion in a splice acceptor site that was only present in B6 genomes after 1976 (Mulligan et al. 2019, 2012). This B6-specific variant causes a global reduction of *Gabra2* transcript and protein expression in brain compared to older B6 strains and to 16 other common and wild-derived inbred mouse strains (Mulligan et al. 2019). Repair of the single nucleotide deletion within the *Gabra2* intron fully restores expression back to the level of other inbred strains (Mulligan et al. 2019). B6 mice with repair of the *Gabra2* allele (Mulligan et al. 2019) provided an ideal platform for testing the modifier potential of *Gabra2* in *Scn1a*^*+/−*^ Dravet mice.

In the current study, we sought to determine if this single nucleotide deletion was responsible for the modifier effect associated with the *Dsm1* locus on chromosome 5. We used B6 mice carrying a repaired (Edited) *Gabra2* allele (Edited/B6 mice) and crossed them with 129.*Scn1a*^*+/−*^ mice to ascertain effects on Dravet phenotypes. We observed rescue of seizure, survival and neuronal phenotypes with this single nucleotide repair, validating *Gabra2* as an epilepsy modifier gene.

## Methods

### Ethics statement

All animal care and experimental procedures were approved by the Northwestern University Animal Care and Use Committee in accordance with the National Institutes of Health Guide for the Care and Use of Laboratory Animals. The principles outlined in the ARRIVE (Animal Research: Reporting of in vivo Experiments) guideline and Basel declaration (including the 3 R concept) were considered when planning experiments.

### Mice

CRISPR/Cas9 editing was used to insert a single intronic nucleotide into *Gabra2* on the C57BL/6J (B6) strain, B6.*Gabra2*^*em1Geh*^, as previously described (Mulligan et al. 2019). Heterozygous edited *Gabra2* mice (Edited/B6) were maintained by continual backcrossing to C57BL/6J (Jackson Labs, #000664, Bar Harbor, ME).

S*cn1a*^*tm1Kea*^ mice with deletion of the first coding exon, were generated by homologous recombination in TL1 ES cells as previously described (Miller et al. 2014). This line has been maintained by continual backcrossing of heterozygotes (abbreviated as *Scn1a*^*+/−*^) to 129S6/SvEvTac inbred mice (129, Taconic, #129SVE, Rensselaer, NY).

To generate double mutant mice, heterozygous Edited/B6 *Gabra2* mice were crossed to 129.*Scn1a*^*+/−*^ mice. The resulting offspring had an overall F1 genome background with B6/129 or Edited/129 alleles at *Gabra2* and WT (+ / +) or heterozygous deletion (+ /−) at *Scn1a*.

Mice were maintained in a Specific Pathogen Free (SPF) barrier facility with a 14-h light/10-h dark cycle and access to food and water ad libitum. Female and male mice were used for all experiments.

### Genotyping

DNA was isolated from P14 tail biopsies using the Gentra Puregene Mouse Tail Kit according to the manufacturer’s instructions (Qiagen, Valencia, CA). For *Gabra2*, approximately 250 ng of DNA was digested with BAMHI-HF (R3136, New England Biolabs, Ipswich, MA) at 37 °C for 1 h. Digested DNA was then diluted 1:1 with nuclease-free water and used as template for droplet digital PCR (ddPCR) using ddPCR Supermix for Probes (No dUTP) (Bio-Rad, Hercules, CA, USA) and a custom TaqMan SNP Genotyping Assay (Life Technologies, Carlsbad, CA) to detect the insertion (Primer 1: 5'-GCTCATCTTTCCATTTTTGCCGAAA; Primer 2: 5'-GGCTTACTACTTCTAAAACATGTACTGTTTTCA; Edited Allele Probe: 5'-GGCTTACTACTTCTAAAACATGTACTGTTTTCA; B6 Allele Probe 2: 5'-FAM-CTATTGTATACTCCTAAATAT-NFQ). Reactions were partitioned into droplets in a QX200 droplet generator (Bio-Rad). PCR conditions were 95 °C for 10 min, then 44 cycles of 95 °C for 30 s and 60 °C for 1 min (ramp rate of 2 °C/sec) and a final inactivation step of 98 °C for 5 min. Following amplification, droplets were analyzed with a QX200 droplet reader with QuantaSoft v1.6.6 software (Bio-Rad). For *Scn1a*, the genotype was determined by multiplex PCR as previously described (Miller et al. 2014).

### Transcript analysis

Whole brain total RNA was extracted from mice with the following *Gabra2* alleles: B6/B6, Edited/B6, B6/129, Edited/129, and 129/129. Total RNA was isolated using TRIzol reagent according to the manufacturer’s instructions. First-strand cDNA was synthesized from 2 µg of RNA using oligo(dT) primer and Superscript IV reverse transcriptase (RT) according to the manufacturer’s instructions (Life Technologies). First-strand cDNA samples were diluted 1:10 and 5 μl was used as template. Quantitative ddPCR was performed using ddPCR Supermix for Probes (No dUTP) (Bio-Rad) and TaqMan Gene Expression Assays (Life Technologies) for mouse *Gabra2* (FAM-MGB-Mm00433435_m1) and *Tbp* (VIC-MGB-Mm00446971_m1) as a normalization standard. Reactions were partitioned into a QX200 droplet generator (Bio-Rad). Thermocycling conditions and analysis were performed as described for genotyping. Both assays lacked detectable signal in no-RT and no template controls. Relative transcript levels were expressed as a ratio of *Gabra2* to *Tbp* concentrations, with 6–13 biological replicates per group. Mice ranged in age from P23 to P41. Statistical comparison between groups was made using ANOVA with Dunnett’s post-hoc comparisons (GraphPad Prism, San Diego, CA). Data are presented as mean ± SEM.

### Immunoblotting

Whole brain P3 membrane protein fractions were isolated from mice with the following *Gabra2* alleles: B6/B6, Edited/B6, B6/129, Edited/129 and 129/129 mice (Hartshorne and Catterall 1984). Membrane fractions (50 μg/lane) were separated on a 7.5% SDS-PAGE gel and transferred to nitrocellulose. Blots were probed with rabbit polyclonal *Gabra2* antibody (822-GA2CL, PhosphoSolutions; RRID:AB_2492101; 1:1000) and mouse monoclonal anti-mortalin/GRP75 antibody (NeuroMab N52A/42; RRID:2120479; 1 μg/mL), which served as a normalization control. Anti-rabbit Alexa Fluor 790 and anti-mouse 680 antibodies (Jackson ImmunoResearch, 1:20,000) were used to detect signal on an Odyssey imaging system (Li-COR). Relative protein levels were determined by densitometry with Image Studio software (Li-COR) and expressed as a ratio of *Gabra2* to GRP75, with 5–6 biological replicates per group. Mice ranged in age from P21 to P42. Statistical comparison between groups was made using one-way ANOVA with Dunnett’s post-hoc comparisons (GraphPad Prism). Data are presented as mean ± SEM.

### Electrophysiology

Acute horizontal hippocampal slices (350 µm) were prepared from 22 to 31-day old male and female littermate mice with B6/B6 (*n* = 3 mice) or B6/Edited (*n* = 3 mice) alleles at *Gabra2*. All mice for this experiment were wild-type at *Scn1a*. Brains were quickly removed after decapitation and sections were made in ice-cold sucrose-slicing artificial cerebrospinal fluid (ACSF) containing the following (in mM): 85 NaCl, 2.5 KCl, 1.25 NaH_2_PO_4_, 25 NaHCO_3_, 25 glucose, 75 sucrose, 0.5 CaCl_2_ and 4 MgCl_2_ with 10 µM DL-APV and 100 µM kynurenate on a Leica Vibratome. Slices were incubated in the same sucrose ACSF for ∼30 min at 30 °C. The solution was gradually exchanged for a recovery ACSF containing the following (in mM): 125 NaCl, 2.4 KCl, 1.2 NaH_2_PO_4_, 25 NaHCO_3_, 25 glucose, 1 CaCl_2_, and 2 MgCl_2_ with 10 µM DL-APV and 100 µM kynurenate at room temperature. Slices were transferred to a recording chamber after a recovery period of at least 1.5 h. Recordings were made from CA1 pyramidal neurons in the hippocampus. Recording electrodes had tip resistances of 3–5 MΩ when filled with internal recording solution containing the following (in mM): 135 CsCl, 20 HEPES, 2 EGTA, 2 Mg-ATP, 0.5 Na-GTP, and 10 QX-314 (pH 7.25). Asynchronous IPSCs (aIPSCs) were recorded from perisomatic synapses where α_2_ GABA_A_ receptor subunits are enriched (Nomura et al. 2019; Prenosil et al. 2006). Perisomatic aIPSCs were recorded in voltage clamp mode (− 70 mV) in the recording strontium-based ACSF containing the following (in mM): 125 NaCl, 2.4 KCl, 1.2 NaH_2_PO_4_, 25 NaHCO_3_, 25 glucose, 6 SrCl_2_, 0.5 CaCl_2_, and 2 MgCl_2_ with blockers of excitatory responses, CNQX (10 µM) and D-APV (50 µM), equilibrated with 95% O_2_ and 5% CO_2_. Perisomatic aIPSCs were evoked by electrical stimuli given through monopolar extracellular stimulating electrodes filled with ACSF and placed in stratum pyramidale and were analyzed within a 50–500 ms window following the stimulus artifact (Fernandes et al. 2015; Jurgensen and Castillo 2015; Nomura et al. 2019; Prenosil et al. 2006). Access resistance (*R*_a_) was continuously monitored and experiments were omitted if the *R*_a_ changed > 20% during the recordings. Four to five cells were recorded per B6/B6 mouse; three to five cells were recorded per Edited/B6 mouse. Statistical comparison between genotype was made using Mann Whitney *U*-Test and paired data were compared using Wilcoxon signed rank test (GraphPad Prism). Data are presented as mean ± SEM.

### 8-Week survival monitoring

*Scn1a*^*+/−*^ littermates with B6/129 or Edited/129 alleles at *Gabra2* were weaned into standard vivarium holding cages containing four to five mice of the same age and sex. Survival was monitored until 8 weeks of age. During that time, all mice were monitored daily for general health. All recorded deaths were sudden and unexpected, occurring in otherwise healthy appearing animals (SUDEP-like). This was the same phenotype used for the original genetic mapping (Miller et al. 2014). Survival was first compared between sexes within genotypes. No sex difference was detected; therefore, groups were collapsed across sex for analysis of genotype effect. Survival statistics were calculated using time-to-event analysis with LogRank Mantel-Cox test (GraphPad Prism).

### Seizure phenotyping

*Scn1a*^*+/−*^ littermate mice carrying B6/129 or Edited/129 *Gabra2* alleles were monitored for spontaneous generalized tonic–clonic seizures (GTCS), as previously described (Hawkins et al. 2017a). Briefly, at P18 or P19, mice were subjected to a single, brief (< 1 min) hyperthermia-induced GTCS and then immediately cooled to baseline temperature. If a GTCS did not occur, the mouse was excluded from the study (< 1%) in order to ensure all *Scn1a*^*+/−*^ mice under study had a similar baseline with an initial ‘priming seizure’. Two to three mice of mixed genotype and sex were placed in a monitoring cage with *ad libitium* access to standard rodent chow and water. Spontaneous GTCS frequency was captured by continuous video monitoring as previously described (Hawkins et al. 2017a, 2016). Mice were monitored beginning at midnight (12–16 h post priming) for 12–14 consecutive days (278–336 h) or until sudden death occurred. This window captures the period of highest seizure frequency in Dravet mouse models (Cheah et al. 2013; Favero et al. 2018; Miller et al. 2014; Mistry et al. 2014; Oakley et al. 2009). Videos were scored offline by reviewers blinded to genotype to determine the frequency and severity of spontaneous GTCS. The total number of seizures for each mouse was divided by the total hours monitored and then converted to a seizure frequency per 24 h. GTCS events were scored using a modified Racine scale adapted for the *Scn1a*^*+/−*^ model as follows: (1) Rearing and paddling, straub tail with no other movement; (2) Rearing and paddling, straub tail, loss of posture, short bursts of movement, often backwards; (3) Rearing and paddling with wild running and/or jumping without loss of posture; (4) Rearing and paddling with wild running and/or jumping with loss of posture; (5) Rearing and paddling with wild running and/or jumping with loss of posture progressing to tonic hindlimb extension (HLE); (6) Rearing and paddling with wild running and/or jumping with loss of posture progressing to tonic HLE ending in death. The number of mice in each score category was compared between groups using a chi-square test. The proportion of seizures with HLE (stage 5–6) was determined for each mouse based on presence or absence of tonic HLE phase for each GTCS event and proportions were averaged by genotype. Seizure frequency and average HLE proportions were first compared between sexes within genotypes. No sex difference was detected; therefore, groups were collapsed across sex for analysis of genotype effect. Seizure frequency and average HLE proportions were compared between genotypes using Mann Whitney U-Tests (GraphPad Prism). Data are presented as means ± SEM.

## Results

**Fig. 1:**
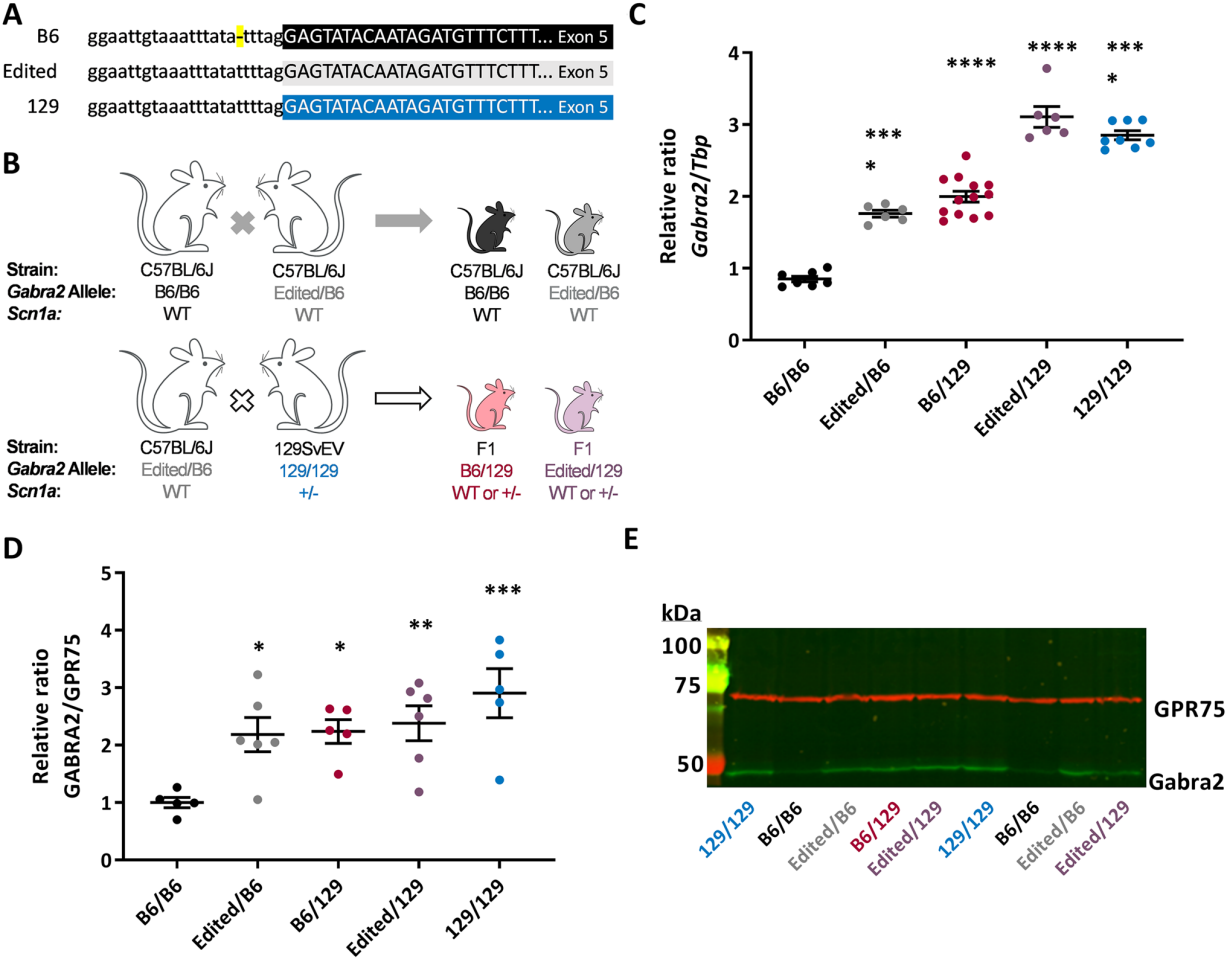
Editing of *Gabra2* B6 allele normalizes expression relative to 129. **A** The B6 *Gabra2* intronic deletion is located on Chr 5 at 71,014,638 bp (GRCm38.p6) (yellow). Sequence of the B6 allele (black) is compared to the Edited (grey) and 129S6/SvEvTac alleles (blue). **B** Breeding scheme for mice used in the study. For expression and electrophysiology experiments, isogenic B6.*Gabra2* Edited mice were crossed with B6 to generate offspring with Edited/B6 (grey) or B6/B6 (black) alleles at *Gabra2*. For expression and seizure experiments, B6.*Gabra2* mice were crossed with isogenic 129.*Scn1a*^*+/−*^ mice to generate F1*.Scn1a*^*+/−*^ or F1. *Scn1a*^+/+^ mice with Edited/129 (purple) or B6/129 (red) alleles at *Gabra2*. **C** Relative expression of *Gabra2* transcript assayed by quantitative RT-ddPCR on whole brain samples from mice with B6/B6, Edited/B6, B6/129, Edited/129 and 129/129 alleles at *Gabra2*. Transcript expression differed between genotypes (*F*_4, 35_ = 104.5, *p* < 0.0001; one-way ANOVA). Relative to B6/B6 (0.85 ± 0.04), *Gabra2* transcript expression was ~ 1.8-fold higher in Edited/B6 (1.78 ± 0.05) and B6/129 (2.00 ± 0.08), and ~ threefold higher in Edited/129 (3.11 ± 0.14) and 129/129 (2.85 ± 0.06). **** *p* < 0.0001, Dunnett’s post-hoc comparisons to B6/B6. Symbols represent samples from individual mice, horizontal lines represent group averages, and error bars represent SEM with 6–13 mice per genotype. **D** Quantification of GABRA2 protein expression determined from western blots of whole brain membrane protein from mice with B6/B6, Edited/B6, B6/129, Edited/129 and 129/129 alleles at *Gabra2* assayed by western blot. Relative GABRA2 protein differed between genotypes (*F*_4, 22_ = 5.349, *p* = 0.0037; One-way ANOVA). Relative to B6/B6 (1.0 ± 0.1), GABRA2 protein expression was ~ 2.2-fold higher in Edited/B6 (2.2 ± 0.3) and B6/129 (2. ± 0.2), and ~ threefold higher in Edited/129 (2.4 ± 0.3) and 129/129 (2.9 ± 0.4). *p < 0.03, **p < 0.01, *** *p* < 0.0009, Dunnett’s post-hoc comparisons to B6/B6. **E** Representative immunoblot probed for GABRA2 (green) and GPR75/Mortalin (red), which served as a loading control

In our prior genetic mapping studies, we nominated *Gabra2* as a top *Dsm1* candidate modifier gene influencing survival of *Scn1a*^*+/−*^ mice and proposed that the observed differential expression of *Gabra2* between 129 and B6 was the likely mechanism (Hawkins et al. 2016). We showed robust strain-dependent differential expression, which was unaffected by genotype (WT v. *Scn1a*^*+/−*^) on the same strain (Hawkins et al. 2019, 2016). Differential expression of *Gabra2* was present prior to seizure onset (P14) and persisted following seizure onset (P24) (Supplemental Fig. S1) (Hawkins et al. 2019). Furthermore, *Gabra2* expression was not altered by recent seizure events (< 24 h before RNA isolation) (Supplemental Fig. S1a) (Hawkins et al. 2019). Expression of other hippocampal GABA_A_ α subunit transcripts (*Gabra1, Gabra3, Gabra4, Gabra5*) did not differ between 129 and B6 or between genotypes (WT v. *Scn1a*^*+/−*^) (Supplemental Fig. S1b) (Hawkins et al. 2019). In parallel work, we attributed lower expression in B6 to a single nucleotide intronic deletion present only in the current B6 genome (Mulligan et al. 2019). Repair of the B6-specific variant by re-insertion of the deleted nucleotide via CRISPR/Cas9 editing restored expression of *Gabra2* to again match that of other mouse strains, including 129. (Fig. 1a) (Mulligan et al. 2019). The B6 mice with restored expression of *Gabra2* (Edited) provide a definitive resource to test the hypothesis that strain-dependent differences in *Gabra2* expression are responsible for the *Dsm1* modifier effect and have even enabled us to localize the effect to specific nucleotide differences among the parental strains.

### Single nucleotide repair of *Gabra2* increases transcript and protein expression

We first confirmed that repair of *Gabra2* (Edited/B6) altered allele-specific expression when crossed with the 129 strain (Fig. 1b). We evaluated transcript and protein expression in wild-type (WT) mice carrying the following alleles at *Gabra2*: B6/B6, Edited/B6, B6/129, Edited/129 or 129/129 (Fig. 1b). *Gabra2* transcript expression differed between genotypes (*F*_4, 35_ = 104.5, *p* < 0.0001, One-way ANOVA) (Fig. 1c). Allele-specific expression differences between B6/B6, 129/B6 and 129/129 were consistent with prior reports (Hawkins et al. 2019, 2016; Miller et al. 2014; Yu et al. 2020). Expression of *Gabra2* transcript in Edited/B6 and B6/129 was elevated ~ 1.8-fold relative to B6/B6 (p < 0.0001, Dunnett’s), while *Gabra2* expression levels in Edited/129 and 129/129 were elevated by ~ threefold relative to B6/B6 (Fig. 1c) (*p* < 0.0001, Dunnett’s). GABRA2 protein expression followed the same pattern as transcript and differed between genotypes, (*F*_4, 22_ = 5.349, *p* = 0.0037, One-way ANOVA) (Fig. 1d, e). GABRA2 expression in Edited/B6 and B6/129 was approximately 2.2-fold higher relative to B6/B6 (p < 0.03, Dunnett’s), while expression in Edited/129 and 129/129 was ~ 2.6-fold higher relative to B6/B6 (*p* < 0.01, Dunnett’s) (Fig. 1d, e). These results demonstrate that repair of the B6 *Gabra2* allele normalized transcript and protein expression to levels that were comparable to 129, consistent with our prior report of normalized expression (Mulligan et al. 2019).

### Single nucleotide repair of *Gabra2* alters neuronal phenotype

**Fig. 2:**
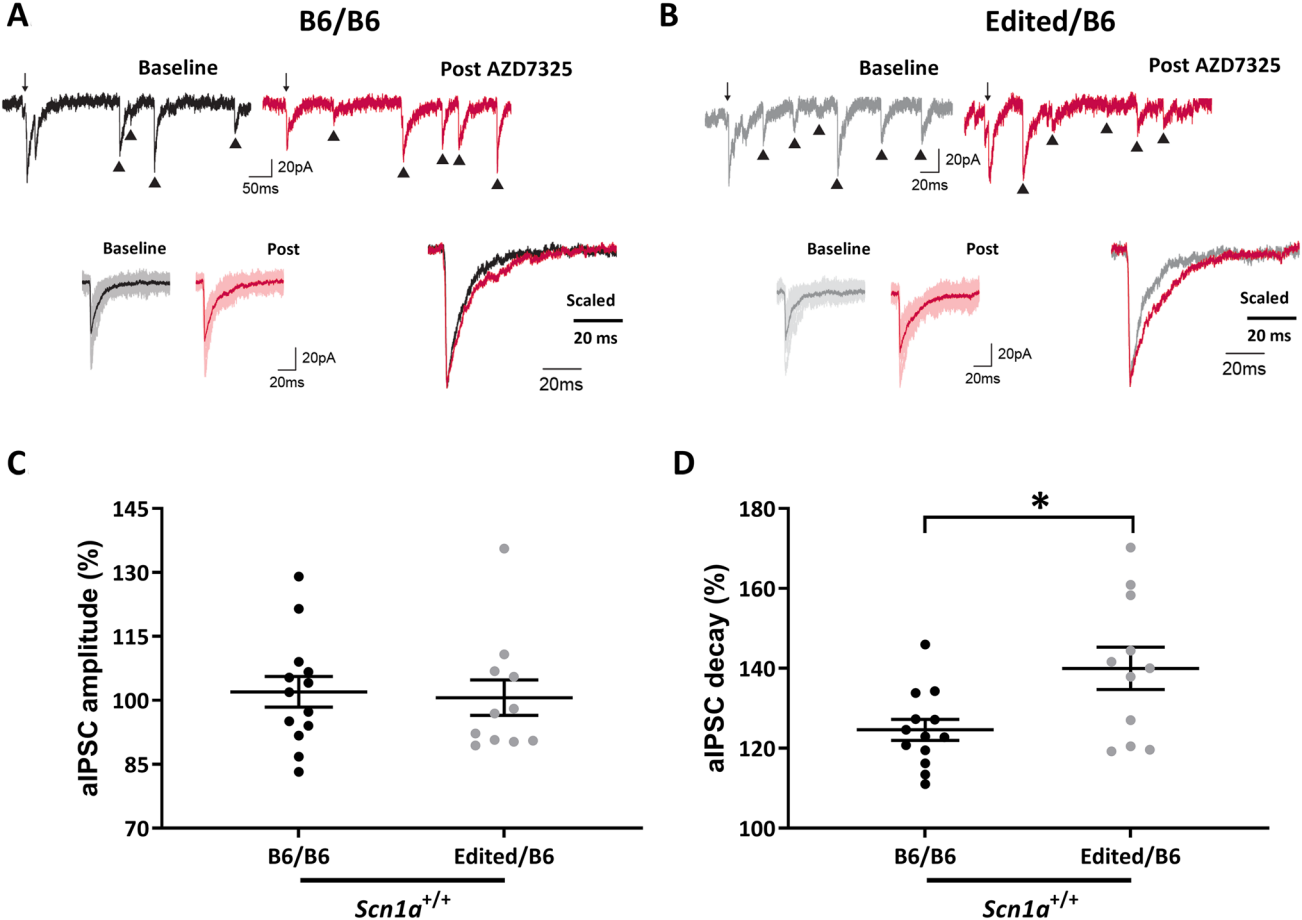
AZD7325 has a larger effect on inhibitory synapses in Edited/B6 mice. **A** Representative traces of perisomatic aIPSCs in CA1 neurons before (baseline) and after AZD7325 (post) treatment in slices from B6/B6 mice and **B** slices from Edited/B6 mice. **C** Effect of AZD7325 on amplitudes of perisomatic aIPSC in CA1 of B6/B6 and Edited/B6 mice. **D** Effect of AZD7325 on the decay kinetics of aIPSC in B6/B6 and Edited/B6 mice. AZD7325 had a greater effect on decay kinetics in Edited/B6 mice (140 ± 5.3%) compared to B6/B6 mice (125 ± 2.6%). *p = 0.047, Mann–Whitney. For panels **C**, **D**, symbols represent samples from individual cells, horizontal lines represent group averages, and error bars represent SEM

We previously demonstrated that perisomatic inhibitory synapses of hippocampal CA1 neurons had a greater abundance of α2-containing GABA_A_ receptors in 129 mice compared to B6 mice (Nomura et al. 2019). α2 GABA_A_ receptor mediated currents can be distinguished by the use of the selective α_2_/α_3_ positive allosteric modulator (PAM) AZD7325, which has a larger effect on slowing the decay kinetics of inhibitory postsynaptic currents (IPSCs) enriched in α2 subunits (Nomura et al. 2019). To determine whether the Edited allele affected synaptic GABA_A_ receptors, we recorded perisomatic IPSCs in CA1 neurons in B6/B6 and Edited/B6 mice. Evoked IPSCs were desynchronized by application of extracellular Sr^2+^ so that asynchronous quantal GABAergic events (aIPSCs) could be recorded. aIPSCs from perisomatic synapses were recorded by stimulating in Stratum Pyramidale (SP) during a control period and after application of AZD7325 (100 nM) and current decay times were measured (Fig. 2). aIPSCs from B6/B6 mice exhibited an average baseline decay of 7.07 ± 0.28 ms during the control period, and aIPSCs from Edited/B6 mice had an average decay of 6.89 ± 0.51 ms during the baseline period. AZD7325 application prolonged aIPSC decay times in slices from both B6/B6 mice (8.76 ± 0.35 ms, *p* = 0.0002, Wilcoxon) and Edited/B6 mice (9.576 ± 0.64 ms, *p* = 0.0010, Wilcoxon). However, the effect of AZD7325 was significantly greater in recordings from Edited/B6 slices, which exhibited a 140 ± 5.3% increase compared to B6/B6 which had a125 ± 2.6% increase (Fig. 2d, *p* = 0.047, Mann–Whitney). This suggests that perisomatic CA1 GABAergic synapses in Edited/B6 mice are enriched in α2-containing receptors compared to those in B6/B6 mice. Consistent with our previous report (Nomura et al. 2019), the amplitude of aIPSC was unaffected by genotype (*p* = 0.12, Mann Whitney) or AZD7325 administration (B6/B6: *p* = 0.94; B6/Edited: *p* = 0.70; Wilcoxon) (Fig. 2c) (B6/B6 baseline: 58.1 ± 3.1 pA, *n* = 13; B6/B6 post: 102 ± 3.6%; and Edited/B6 baseline: 68.5 ± 4.7 pA, *n* = 11; Edited/B6 post: 100.6 ± 4.2%). Furthermore, aIPSC frequency (B6/B6 baseline: 6.56 ± 0.56 Hz; B6/B6 post: 6.92 ± 0.44 Hz; and Edited/B6 baseline: 7.10 ± 0.87 Hz; Edited/B6 7.37 ± 0.59 Hz) was unaffected by genotype (*p* > 0.999, Mann–Whitney) or AZD7325 administration (B6/B6 *p* = 0.33, Edited/B6 *p* = 0.50, Wilcoxon). These results demonstrate that repair of the B6-specific *Gabra2* allele normalized functional expression of α2-containing GABA_A_ receptors in perisomatic CA1 GABAergic synapses, likely by altering subunit composition rather than altering the number of perisomatic GABA_A_ receptors.

### Single nucleotide repair of *Gabra2* improves phenotype of F1.*Scn1a*^*+/−*^ mice

**Fig. 3:**
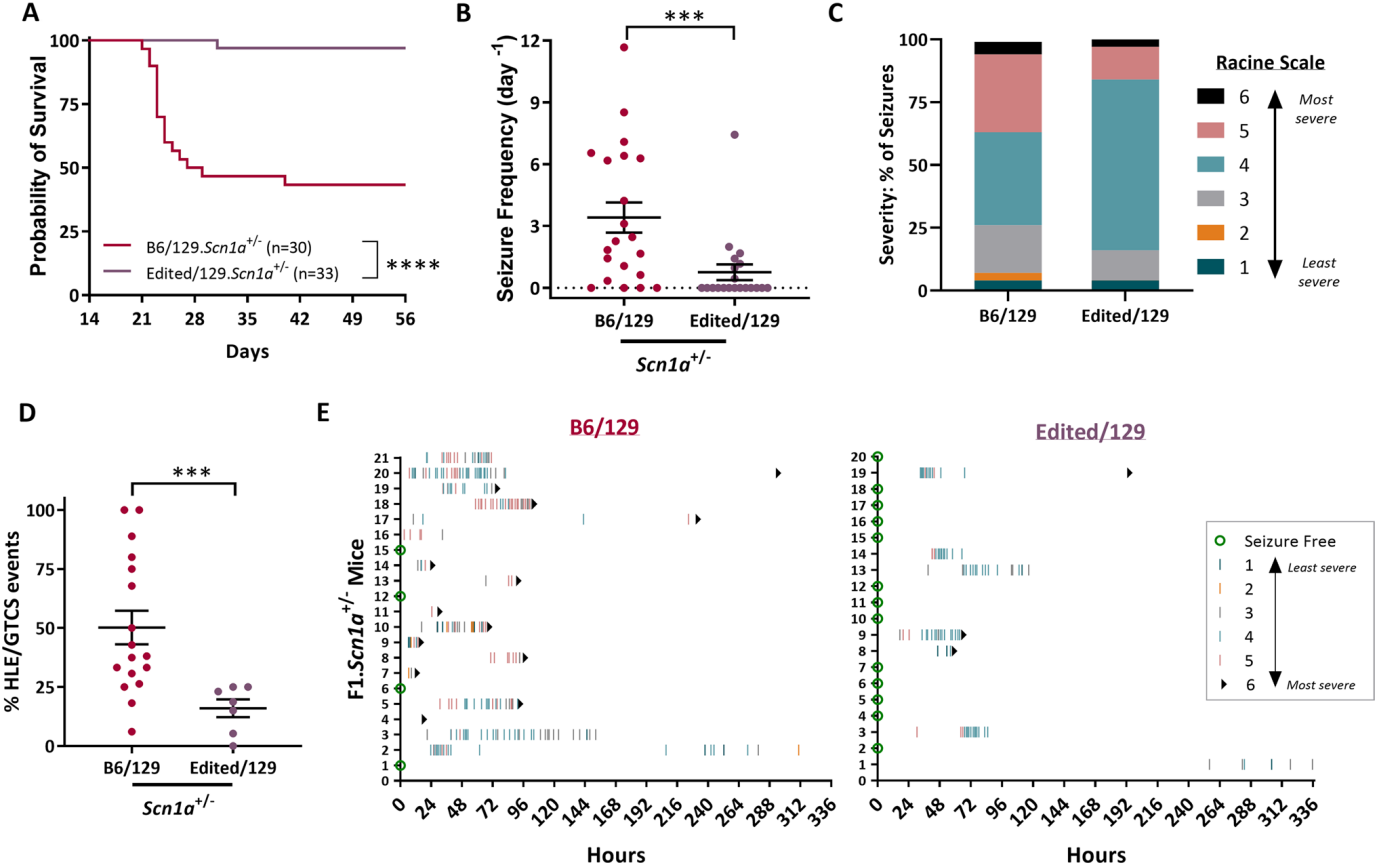
Survival and seizure burden improved in F1.*Scn1a*^*+/−*^ with Edited/129 versus B6/129 alleles at *Gabra2*. **A** Kaplan Meier survival plot comparing 8 week survival of B6/129 and Edited/129 F1.*Scn1a*^*+/−*^ mice. Survival was worse in B6/129 mice (43%) compared to Edited/129 (97%) with *n* = 30–33 per genotype. ****p < 0.0001, Logrank Mantel-Cox. **B** The proportion of mice exhibiting spontaneous GTCS and average GTCS frequency differed between F1.*Scn1a*^*+/−*^ mice with B6/129 (81% with seizures; 3.4 ± 0.7 GTCS/day) versus Edited/129 (35% with seizures; 0.8 ± 0.4 GTCS/day) alleles at *Gabra2*. Symbols represent samples from individual mice, horizontal lines represent the group average, and error bars represent SEM with *n* = 20–21 per genotype. ***p = 0.0008, Mann–Whitney. **C** Percentage of seizures in each category of an adapted modified Racine scale (colors defined below) differed between F1.*Scn1a*^+/−^ mice with B6/129 or Edited/129 alleles at *Gabra2*. The majority of seizures in Edited/129 mice scored ≤ 4 compared to 63% in B6/129 mice. B6/129 *n* = 246 seizures total; Edited/129 *n* = 94 seizures total. *p* < 0.0006, chi-square test. **D** Among mice with GTCS, the average proportion of GTCS that progressed to HLE differed between mice with B6/129 (0.50 ± 0.07) versus Edited/129 (0.16 ± 0.04) alleles at *Gabra2*. Symbols represent samples from individual mice, and error bars represent SEM with *n* = 7–17 per genotype. ***p = 0.0007, Mann–Whitney. **E** Spontaneous GTCS diary plots for individual F1.*Scn1a*^*+/−*^ mice with B6/129 or Edited/129 alleles at *Gabra2*. Each row represents a single F1.*Scn1a*^*+/−*^ mouse (*n* = 20–21 per genotype) over the 336-h monitoring period or until occurrence of sudden, unexpected death indicated by a black triangle. Green circles represent subjects with no GTCS events. Tick marks represent individual seizure events with color indicating severity score using an adapted modified Racine scale. Colors used for the modified Racine scale adapted for *Scn1a*^*+/−*^ mice in **c** and **e** are as follows: Green = 1, rearing and paddling, straub tail with no other movement; Orange = 2, rearing and paddling, straub tail, loss of posture, short bursts of movement, often backwards; Grey = 3, rearing and paddling with wild running and/or jumping without loss of posture; Teal = 4, rearing and paddling with wild running and/or jumping with loss of posture; Pink = 5, rearing and paddling with wild running and/or jumping with loss of posture progressing to tonic hindlimb extension (HLE); Black = 6, rearing and paddling with wild running and/or jumping with loss of posture progressing to tonic hindlimb extension (HLE) and death

129.*Scn1a*^*+/−*^ mice have no overt unprovoked seizures or premature lethality phenotype, whereas F1.*Scn1a*^*+/−*^ mice have spontaneous seizures and premature lethality (Kang et al. 2018; Miller et al. 2014; Ogiwara et al. 2007; Yu et al. 2006). Similarly, mapping with interval-specific congenic (ISC) strains demonstrated that homozygosity for 129 alleles in the *Gabra2* region could rescue seizure and sudden death phenotypes in otherwise F1.*Scn1a*^*+/−*^ mice (Hawkins et al. 2016). To further refine the genetic mechanism, we investigated whether repair of the B6-specific *Gabra2* intronic variant could improve survival of F1.*Scn1a*^*+/−*^ mice. We crossed B6 mice with heterozygous repair of *Gabra2* (B6/Edited) to 129.*Scn1a*^*+/−*^ to generate F1.*Scn1a*^*+/−*^ mice carrying Edited/129 or B6/129 alleles at *Gabra2* (Fig. 1b). Survival was improved in F1.*Scn1a*^*+/−*^ mice with Edited/129 versus B6/129 alleles at *Gabra2* (*p* < 0.0001, Logrank Mantel-Cox). Mice with Edited/129 alleles had 97% (32 of 33) survival to 8 weeks of age compared to only 43% (13 of 30) of mice with B6/129 alleles (Fig. 3a), supporting *Gabra2* as the modifier gene at the *Dsm1* locus, which was originally mapped using 8-week survival as the primary phenotype (Miller et al. 2014).

Next, we investigated if the *Gabra2* variant altered seizure frequency and/or severity. At P18-19, F1.*Scn1a*^*+/−*^ mice with Edited/129 and B6/129 alleles at *Gabra2* were subjected to a single hyperthermia-induced priming seizure and quickly cooled to baseline temperature (Hawkins et al. 2017a). We previously demonstrated that this paradigm enhances seizure incidence in F1.*Scn1a*^*+/−*^ mice and improves discrimination power for ascertaining seizure reduction (Hawkins et al. 2017a, 2017b). Importantly, the temperature for seizure onset did not differ between Edited/129 (41.1 ± 0.2 °C) and B6/129 (41.2 ± 0.2 °C) groups (*p* = 0.9127, Student’s *t*-test), as expected since 129.*Scn1a*^*+/−*^ mice also have a similar temperature threshold for hyperthermia-induced seizures (Supplemental Fig. S2). All mice in both the Edited/129 and B6/129 groups had a hyperthermia-induced priming seizure. Following the priming seizure, mice were continuously monitored for subsequent spontaneous generalized tonic–clonic seizures (GTCS) for 12–14 days or until sudden death occurred. This window coincides with the period of highest seizure frequency in Dravet mouse models (Cheah et al. 2013; Favero et al. 2018; Miller et al. 2014; Mistry et al. 2014; Oakley et al. 2009). The proportion of F1.*Scn1a*^*+/−*^ exhibiting GTCS during the monitoring period differed between *Gabra2* genotypes (*p* < 0.0026, Fisher’s exact). Only 35% of mice with Edited/129 alleles exhibited seizures, while 81% of mice with B6/129 alleles had seizures (Fig. 3b). Seizure frequency in F1.*Scn1a*^*+/−*^ mice with Edited/129 alleles was lower (0.8 ± 0.4 GTCS/day) relative to mice with B6/129 alleles (3.4 ± 0.7 GTCS/day) (Fig. 3b) (*p* < 0.0018, Mann–Whitney).

While previous studies suggested a correlation between seizure frequency and survival, additional studies have shown that the relationship between total seizure frequency and survival is more complex, and that seizure severity is a critical factor (Kalume et al. 2013; Teran et al. 2019). Recorded/witnessed sudden death events in *Scn1a*^*+/−*^ mice occur exclusively following seizures that progress to tonic HLE, while high frequencies of non-HLE seizures are tolerated (Hawkins et al. 2017a, 2017b; Kalume et al. 2013; Kang et al. 2019; Teran et al. 2019). Therefore, we also assessed seizure severity using a modified Racine scale adapted for the *Scn1a*^*+/−*^ Dravet model. Across the population of mice with Edited/129 alleles, 84% of seizures scored 4 or below, while 63% of seizures in mice with B6/129 alleles scored similarly. Importantly, the proportion of GTCS events progressed to HLE in individual mice with Edited/129 alleles was lower (16 ± 4% of events) relative to mice with B6/129 alleles (50 ± 7% of events) (*p* = 0.0007, Mann–Whitney) (Fig. 3c, d). Seizure diary plots show individual mice and their respective scored seizure events (Fig. 3e). Together, these data demonstrate that repair of the B6 *Gabra2* allele lessened seizure burden of F1.*Scn1a*^*+/−*^ mice, supporting *Gabra2* as the gene responsible for the modifier effect associated with the *Dsm1* locus.

## Discussion

In the present study, we demonstrated that *Gabra2* is the modifier gene *Dsm1* responsible for the strain-dependent difference in survival between F1 and 129 *Scn1a*^*+/−*^ mice attributed to *Dsm1* (Hawkins et al. 2016; Miller et al. 2014). Furthermore, we defined the responsible nucleotide difference underlying the modifier effect. Editing of the B6-specific single nucleotide intronic deletion in *Gabra2* normalized brain transcript and protein expression relative to the 129 allele, elevated enrichment of α2-containing GABA_A_ receptors in hippocampal synapses, and dramatically improved seizure and sudden unexpected death (SUDEP-like) phenotypes in the F1.*Scn1a*^*+/−*^ Dravet mouse model. This work has clear therapeutic implications and suggests that interventions that increase CNS expression or function of GABRA2 should improve outcomes in Dravet syndrome. Moreover, *Gabra2* was also recently nominated as a modifier of survival based on genetic mapping in a mouse model of *SCN8A*-associated DEE (Yu et al. 2020). Thus, the therapeutic implications may apply more broadly to other DEEs.

In previous work we and others demonstrated robust allele-specific expression of *Gabra2* (Hawkins et al. 2019, 2016; Mulligan et al. 2019, 2012; Yu et al. 2020). Also, we established that unusually low GABRA2 protein expression is caused by a non-coding single nucleotide deletion in the C57BL/6J genome that alters splicing efficiency (Hawkins et al. 2017b; Korostynski et al. 2006; Mulligan et al. 2019, 2012; Yeo et al. 2016). B6 mice are the most commonly used laboratory mouse strain and was the first to be sequenced (GRCm38). With drastic improvements in next-generation sequencing techniques, it has become evident the GRCm38 reference genome, generated from filial (F) generation 204–207 mice, may differ from the current (~ F226) B6 mouse strain (Fairfield et al. 2015; Sarsani et al. 2019). Recently, Sarani et al. sequenced C57BL/6JEve, the “mother” (F223) of the current laboratory B6 animals sourced from the Jackson Laboratory (F226) (Sarsani et al. 2019). The group identified 59 indels and inversions between B6-Eve and GRCm38, many located within noncoding intronic regions (Sarsani et al. 2019). Furthermore, recent efforts from the Jackson Laboratory identified 1083 quality control-filtered variants between the GRCm38 reference sequence and sequencing from the most recent inbreeding generations of C57BL/6J (Fairfield et al. 2015). These variants can provide insight into the private de novo variants in the B6 genome compared to other inbred strains. This is of particular interest when investigating genetic modifiers of diseases, including epilepsy. Another recent example of a B6-specific de novo variant that acts as a genetic modifier of epilepsy is the C50T variant in neuronal tRNA n-Tr20. The C50T variant results in lower n-Tr20 levels that are associated with enhanced seizure susceptibility in WT mice and elevated spike wave discharge incidence in the *Gabrg2*^*R43Q*^ absence epilepsy mouse model (Ishimura et al. 2014; Kapur et al. 2020).

While editing of the C57BL/6J-specific *Gabra2* allele improved many aspects of the Dravet-like phenotype, F1.*Scn1a*^*+/−*^ characteristics were not diminished into a benign 129.*Scn1a*^*+/−*^ phenotype, as spontaneous seizures were still observed. This was not unexpected as our initial genetic mapping of the Dravet survival modifiers (*Dsm*) identified multiple loci (*Dsm1-5*), with the protective effect of 129 alleles at the *Dsm1* locus accounting for 4–10% of the phenotypic variance (Miller et al. 2014). In terms of *Dsm1*, the magnitude of *Gabra2* allele effects was similar to our prior fine mapping study using interval-specific congenic mice, with survival improving to 98 or 92%, respectively, and 55 or 42% reduction in occurrence of severe HLE seizures (Hawkins et al. 2016). This suggests that *Gabra2* is the major modifier at the *Dsm1* locus. Three additional modifier loci of the *Scn1a*^*+/−*^ phenotype (*Dsm2*, *Dsm3*, *Dsm5*) have yet to be investigated (Miller et al. 2014). Preliminary examination of B6-specific private variants may suggest additional candidate modifier genes of survival in the mouse model of Dravet syndrome. Furthermore, our prior RNA-seq study demonstrated that strains differ in their homeostatic response to the *Scn1a*^*+/−*^ mutation, and we and others have shown that seizures themselves can induce additional homeostatic remodeling (Dutton et al. 2017; Favero et al. 2018; Hawkins et al. 2017a, 2019; Salgueiro-Pereira et al. 2019).

**Fig. 4:**
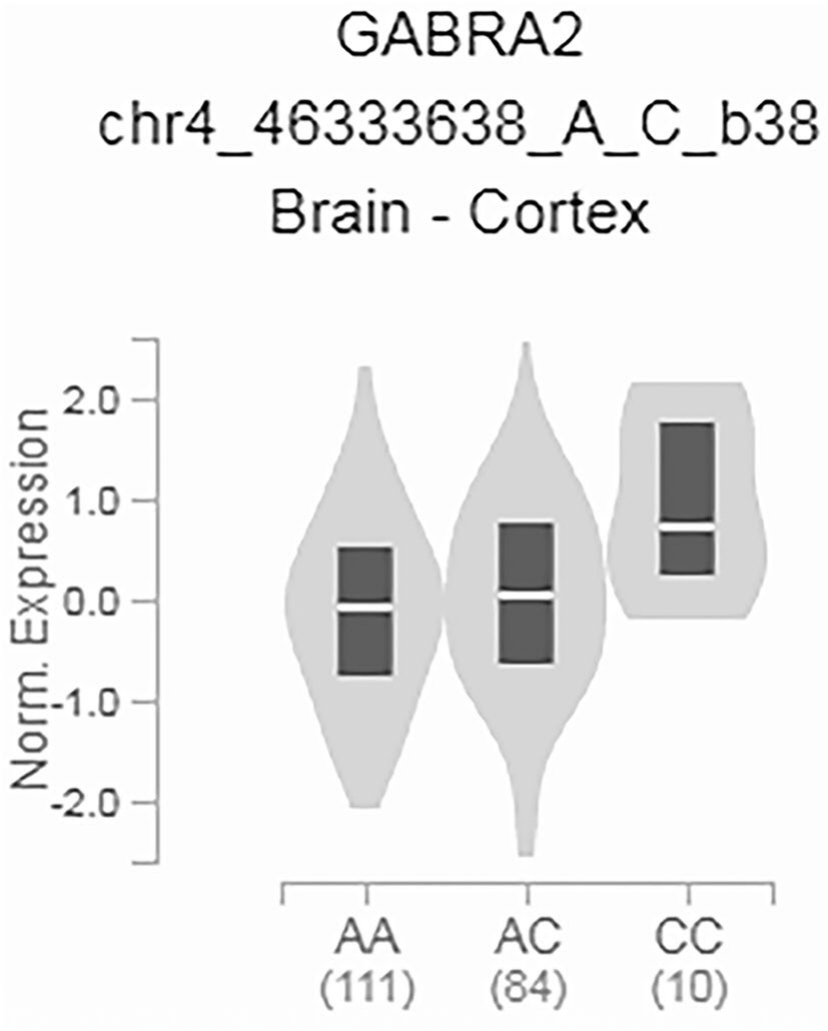
Human *GABRA2* eQTL. Genetic variation at SNP rs279829 (chr4:46,333,638 in GRCh38.p12) is associated with variation in *GABRA2* transcript expression (*p* = 0.0000025; *n* = 205 donor samples). Individuals that are homozygous for the minor allele have elevated expression relative to individuals that are heterozygous or homozygous for the major allele. Data used for this figure was obtained from: the GTEx Portal on 07/16/2020 (dbGaP Accession phs000424.v8.p2) [https://www.gtexportal.org/home/gene/GABRA2#eQTLBlock]

A potential limitation of this study is that the single nucleotide deletion in B6 is a mouse-specific variant, which is not present in humans. However, studies on human tissue have identified multiple eQTLs for *GABRA2*, where expression level varies in association with genetic variation. For example, the single nucleotide polymorphism rs279829 has an overall minor allele frequency (MAF) of 0.2325, varying among populations with a MAF of 0.5259 in East Asian populations compared to a MAF of 0.05706 in African populations (Karczewski et al. 2020). Homozygous carriers of the minor allele have elevated *GABRA2* expression relative to individuals that are heterozygous or homozygous for the major allele (Fig. 4) (GTEx Consortium 2015). Thus, natural variation in *GABRA2* expression may contribute to variable expressivity of Dravet syndrome and other epilepsy phenotypes, and possibly contribute to treatment response for drugs that target GABA_A_ receptors. Although we did not define the precise mechanism whereby restoration of α2 containing receptors alters inhibitory balance, it is likely that enhanced perisomatic phasic inhibition is predominantly mediated by α2-containing receptors, which is supported by the differential effect of AZD7325 in B6/edited mice. The lack of difference in the baseline properties of IPSC in B6/edited mice does not exclude this possibility. IPSC amplitude and kinetics are not altered even when the α2 subunit is completely knocked out (Panzanelli et al. 2011). This indicates that the majority of GABA_A_ receptors at these synapses are heteromeric combinations of the α1 and α2 subunits, and the α1 subunit is dominant in dictating the properties of synaptic currents. Consistent with our previous study (Nomura et al. 2019), AZD7325 treatment had a greater effect in prolonging the decay kinetics of perisomatic IPSCs without affecting the amplitude, demonstrating a differential contribution of α2 activity in these synapses in B6/edited mice.

Interestingly, although there is strong strain-dependence for spontaneous seizures and premature death, strain-dependence does not extend to hyperthermia-induced seizures as *Scn1a*^*+/−*^ mice on F1 and 129 strains have similar threshold temperatures. This suggests that 129 alleles shift seizure risk, rather than providing complete protection, and that hyperthermia is a strong provocateur in the context of an *Scn1a* mutation. This may have implications for preclinical screening assays, as hyperthermia may be a more demanding condition relative to spontaneous seizure monitoring.

This study highlights the importance of continued efforts in identifying modifier genes in mouse models. Several epilepsy modifier genes, including *CACNA1G* and *GABRA2*, were first identified in the *Scn1a*^*+/−*^ mouse model of Dravet syndrome and later confirmed as epilepsy risk genes in humans (Bergren et al. 2005, 2009; Butler et al. 2018; Calhoun et al. 2016, 2017; Chemin et al. 2018; Feng et al. 2019; Hawkins and Kearney 2012, 2016; Hawkins et al. 2011, 2016; Hernandez et al. 2016; Kearney et al. 2006; Miller et al. 2014). Identifying modifier genes can provide refined insights into the molecular basis of genetic disease. Furthermore, it may provide the basis for improving predictions about disease course and clinical management. Finally, modifier genes and pathways can provide novel targets for therapeutics. Previously, we used AZD7325, a GABA_A_ α2/α3-selective PAM, to modulate the neuronal phenotype of *Scn1a*^*+/−*^ mice and demonstrated protective effects against hyperthermia-induced seizures (Nomura et al. 2019). The current study confirms that AZD7325 can be used to distinguish the GABA_A_ receptor type in perisomatic synapses and directly demonstrates that the Edited allele contributes to an enriched α2 subunit content. Future studies assessing efficacy of treatment with AZD7325 or other GABRA2-selective PAMs on spontaneous seizures and survival would provide further support for targeting α_2_-containing GABA_A_ receptors for the treatment of Dravet syndrome and, potentially, other DEEs that share reduced GABAergic signaling as a common pathogenic mechanism.

## Supplementary Information

Below is the link to the electronic supplementary material.

Supplementary file1 (PDF 595 kb)

## Data Availability

All data and material are available upon reasonable request.
