# Supplementary material for: Gabra2 is a genetic modifier of Dravet syndrome in mice
Source: Mamm Genome. 2021 Jun 4;32(5):350–63. doi: 10.1007/s00335-021-09877-1 (PMC8458207; doi:10.1007/s00335-021-09877-1)
Supplement: Supplementary file 1 — Supplementary file1 (PDF 595 kb) [file 335_2021_9877_MOESM1_ESM.pdf]

Supporting Information for

Gabra2 is a genetic modifier of Dravet syndrome in mice

Nicole A. Hawkins[1], Toshihiro Nomura[2], Samantha Duarte[1], Levi Barse[1], Robert W. Williams[3], Gregg E. Homanics[4], Megan K. Mulligan[3], Anis Contractor[2,5] and Jennifer A. Kearney[1*]

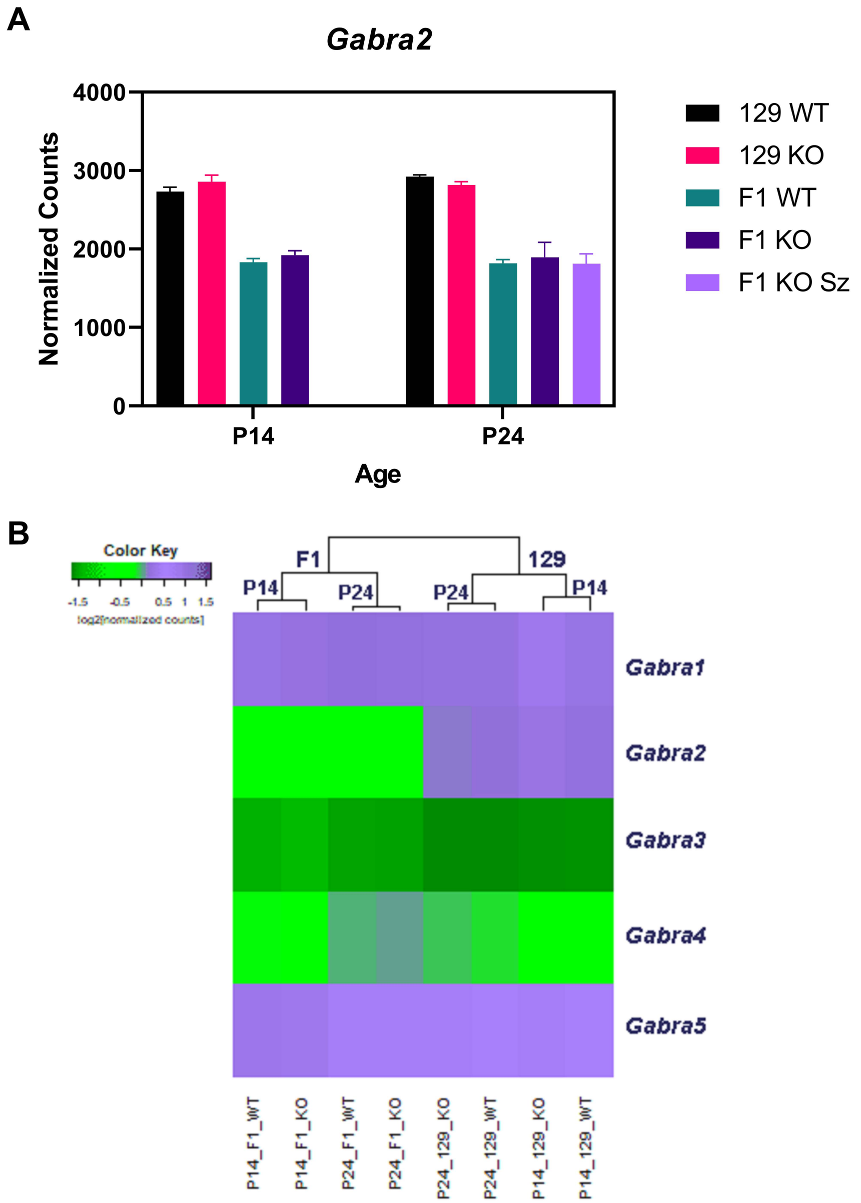

Supplemental Figure S1. Differential expression of *Gabra2* in WT and *Scn1a*$^{+/-}$ mice prior to (P14) and following onset of seizures (P24). **(A)** Strain-specific expression differences in hippocampal *Gabra2* expression by RNA-seq [1]. *Gabra2* transcript expression differed between the 129 and B6 strains at P14 for both WT (FDR-adj P-value=$7.5e^{-18}$) and *Scn1a*$^{+/-}$ (KO) mice (FDR-adj P-value=$7.1e^{-17}$), as well as at P24 for both WT (FDR-adj P-value=$1.1e^{-38}$) and *Scn1a*$^{+/-}$ (FDR-adj P-value=$2.5e^{-7}$). Occurrence of a recent seizure (<24 hours prior to RNA isolation), did not change the level of *Gabra2* expression, with no difference in expression between P24 *Scn1a*$^{+/-}$ mice with (F1 KO Sz) or without a recent seizure (F1 KO) (FDR-adj P-value=0.913082391). **(B)** Heatmap of $GABA_A$ subunits transcript expression in hippocampus in WT and *Scn1a*$^{+/-}$ at P14 and P24. Expression of *Gabra2* differed between strains as detailed in (A), while transcript expression of other $GABA_A$ subunits did not significantly differ between strains. Significance was considered as a FDR-adjusted P-value threshold <0.05 and ≥1.5 log-fold change. All data available in supplemental tables from Hawkins et al, 2019 [1].

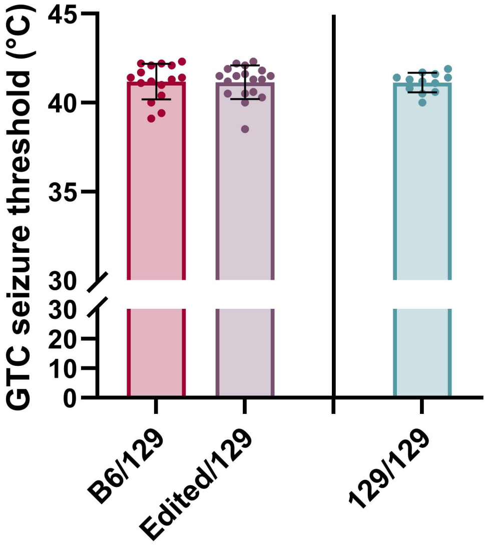

Supplemental Figure S2. Temperature thresholds for hyperthermia-induced seizures in *Scn1a*$^{+/-}$ mice does not differ across strain backgrounds. Threshold temperatures for GTCS induced by hyperthermia for F1.*Scn1a*$^{+/-}$ mice with B6/129 or Edited/129 alleles at *Gabra2* (this study) or 129.*Scn1a*$^{+/-}$ mice (separate cohort). F1.*Scn1a*$^{+/-}$ mice with B6/129 or Edited/129 alleles at *Gabra2* exhibited subsequent spontaneous seizures in the week following hyperthermia, while 129.*Scn1a*$^{+/-}$ mice did not. Symbols represent threshold temperatures of individual mice and bars show average temperatures per genotype group. Error bars represent standard deviation, with n = 12–18 per group. ($F_{2,43}$ = 0.01507, p=0.9850; one-way ANOVA)

References

1. Hawkins NA, Calhoun JD, Huffman AM, Kearney JA. Gene expression profiling in a mouse model of Dravet syndrome Exp Neurol. 2019 Jan;311:247-256.
